# The Distribution of Exon 3-Deleted/Full-Length Growth Hormone Receptor Polymorphism in the Turkish Population

**DOI:** 10.4274/jcrpe.v3i3.25

**Published:** 2011-09-09

**Authors:** Firdevs Baş, Fatih Keleşoğlu, Özlem Timirci, Sema Kabataş Eryılmaz, Nilüfer Bozkurt, Banu Küçükemre Aydın, Rüveyde Bundak, Turgay İsbir, Feyza Darendeliler

**Affiliations:** 1 Istanbul University Istanbul Faculty of Medicine, Pediatric Endocrinology Unit, Istanbul, Turkey; 2 Istanbul University Institute of Experimental Medicine, Molecular Medicine Unit, Istanbul, Turkey; +90 212 533 13 83 firdevsb@istanbul.edu.tr

**Keywords:** Human growth hormone, growth hormone receptor, genetic polymorphism

## Abstract

**Objective:** The exon 3-deleted/full-length (d3/fl) growth hormone receptor (d3/fl-GHR) polymorphism has been associated with responsiveness to GH therapy in some children and also with adult height variation in the general population. We aimed to evaluate the distribution of d3/fl-GHR polymorphism in a Turkish population.

**Methods:** The study included 477 (54 females/423 males) healthy adults with a mean±SD age of 31.1±9.0 years (range: 18-57). Height and body mass index (BMI) were expressed as standard deviation score (SDS) according to national standards. All adults had normal height and BMI SDSs (between -2 and +2). GHR exon 3 isoforms were studied by simple multiplex polymerase chain reaction method. Insulin-like growth factor-1 (IGF-1) and IGF-binding protein-3 (IGFBP-3) values were also  measured and expressed as SDS.

**Results:**  The distribution of the GHR exon 3 genotypes in the Turkish  healthy adults was  35% (n=167) for fl/fl, 39% (n=186) for fl/d3, and 26% (n=124) for d3/d3. There was no difference between genders in GHR exon 3 genotypes. Frequencies of fl allele  and d3 allele were  54.5% and 45.5%, respectively. There were no differences in height SDS and BMI SDS among the three d3/fl-GHR genotype groups. There was a significant difference in IGFBP-3 SDS between fl/fl and fl/d3 groups (p=0.022).

**Conclusions:** This study presents the results of GHR polymorphism in a Turkish population as a reference for further studies. The distribution was similiar to European populations. There were no correlations between GHR isoforms and height SDS or other clinical/biochemical characteristics of the individuals except for higher IGFBP-3 levels in the fl/d3 group as compared to the  fl/fl group. Whether this finding implies an abnormality, needs further investigation.

**Conflict of interest:**None declared.

## INTRODUCTION

The growth hormone insulin like growth factor-1 (GH-IGF-1) axis is the key regulator of somatic growth in humans. Adult height is controlled by genetic and non-genetic factors. Common variants in genes of  the GH-IGF-1 axis  contribute to height variation in the general population (1). Genetic variations in the GH-IGF-1 axis might also affect the response to GH treatment.  GH action is mediated through the activation of its surface receptor, GH receptor (GHR), a 620 amino acid single-transmembrane protein. The binding of GH to GHR results in the formation of a receptor homodimer. Interaction of dimerized GHR with intracellular tyrosine kinase JAK-2 leads to the phosphorylation of downstream signal transduction molecules, induction of signal transducers and activators of transcription (STAT) proteins, and stimulation of mitogen-activated protein (MAP) kinases. Activated STAT5 is then translocated to the nucleus where it transactivates the expression of IGF-1 and other GH-dependent genes (2,3,4). 

GHR gene is located on the short arm of chromosome 5 (p13.1-p12) (5,6). It consists of  nine coding exons (exon 2-10) and several untranslated exons; exon 2 codes for the signal peptide, exon 3-7 encode the extracellular domain, exon 8 encodes the transmembrane domain, and exon 9-10 encode the cytoplasmic domain (5). GHR polymorphisms may affect the response to exogenous GH treatment in patients with short stature due to differences in GH pharmacogenetics. Polymorphisms in the GHR gene have been described for exons 3, 6 and 10 (7). Two of the most common GHR isoforms in humans are generated by retention (fl-GHR) or deletion (d3-GHR) of exon 3, which encodes a sequence in the extracellular domain. In the Caucasian population, the frequency of fl-GHR has been estimated to range from 56% to 75% and that of d3-GHR from 25% to 44% (8,9,10,11,12). In Asian populations,  d3-GHR allele was found to be less frequent (13,14,15). 

 The aim of this study was to evaluate the distribution of  GHR exon 3 polymorphisms in the Turkish population. 

## MATERIALS AND METHODS

The study included 477 (54 females/423 males) healthy adults. The volunteers were recruited from blood donors and hospital staff. Mean±SD age was 31.1±9.0 years (range: 18-57). None of the subjects had received therapy with GH or any other anabolic drug. A single subject per family was included in the study. Height and weight were measured by standard methods and body mass index (BMI) was calculated as weight (kg)/height (m)2.  Height, weight and BMI were expressed as standard deviation score (SDS) (16,17). Height and BMI SDSs were within normal ranges (between - 2 and + 2 SD) in all subjects.  Blood samples were taken from all subjects for measurement of  IGF-1 and IGF binding protein-3 (IGFBP-3) and for isolation of DNA.

  IGF-1 (ng/mL) was measured by IRMA method  (DSL-5600, Webster, Tx). The limit of sensitivity was 0.80 ng/mL. Intra and inter-assay coefficient of variation (CV)  values  were 1.5-3.4% and 1.5-8.2%, respectively. IGFBP-3 (ng/mL), also measured by IRMA method (DSL- 6600, Webster, Tx), has a limit of sensitivity of 0.5 ng/mL.  Intra and inter-assay CV were 1.8–3.9% and 0.5–1.9%, respectively. IGF-1 and IGFBP-3 SDSs were calculated using reference values of the IGF-1 and IGFBP-3 assays (http://www.beckmancoulter.com).  

Subanalysis was carried out by grouping the patients according to their height SDS as the tallest (+2 and >+1), the average (≤+1 and >-1), and the shortest (≤-1 and ≥-2) height groups.

**Molecular Analysis**

Genomic DNA was isolated from peripheral blood  leucocytes of healthy adults by DNA isolation kit (MagNA pure kit, Roche Diagnostics, Mannheim, Germany) in Istanbul Faculty of Medicine at Istanbul University. 

The frequency of the GHR transcript variant [full length (fl) or exon 3 deleted (d3)] was tested by a simple multiplex polymerase chain reaction (PCR) assay, using one sense primer (G1) and two antisense primers one specific for GHRfl (G3) and the other specific for GHRd3 isoform (G2) (GenBank accession number AF155912), as described by Pantel et al (8). Amplification products were analyzed by electrophoresis on 1% agarose gel stained with ethidium bromide. The fl allele is represented by a 935-bp fragment and the d3 allele by a 532-bp fragment. A second PCR assay for samples initially genotyped as homozygous GHRd3 (d3/d3), using the primer specific to the GHRfl  isoform (G1 and G3), was performed to avoid false  homozygous GHRd3 genotyping.

**Ethics**

This study was approved by the local ethics committee. Written informed consent was obtained from all subjects. 

**Statistical Analysis**

Statistical analyses were done using SPSS version 12.0 (SPSS Inc., Chicago, IL). Values are expressed as mean±SD. Comparison between the means was done by parametric tests. Height SDS in the healthy adults was analyzed for normal distribution by the Kolmogorov-Smirnov test (c2=0.085, p=0.000). Hardy-Weinberg equilibrium (HWE) was calculated according to standard procedures using c2 analysis.  Differences between the variables evaluated among the d3/fl-GHR genotypes were calculated using ANOVA (Bonferroni). Differences for the d3/fl-GHR genotype frequencies were analyzed by the c2 test. A p-value of less than 0.05 was considered statistically significant. 

## RESULTS

The distribution of the three GHR exon 3 genotypes in this Turkish healthy adult population was  35% (n=167) for fl/fl, 39% (n=186) for fl/d3, and 26% (n=124) for d3/d3. There was no difference between genders with respect to the distribution of these three GHR exon 3 genotypes. There were also no differences in height and BMI SDSs among the three d3/fl-GHR genotype groups. IGFBP-3 SDS was significantly higher in the fl/d3 group as compared to the fl/fl group (p=0.022) (Table 1). 

Genotype frequencies reached HWE only in the tallest height group, but not in the average and the shortest height groups. In the total population, frequencies of fl allele  and d3 allele were  54.5% (n=520) and 45.5% (n=434), respectively. Allele frequencies were similar in the three height groups. There were no differences in genotypes or in IGF-1 and IGFBP-3 SDS values among subjects grouped according to their height SDS. IGF-1 SDS was found to be higher in the tallest height group, but this difference was not significant (Table 2).  There was a positive significant correlation between BMI SDS and IGFBP-3 SDS  in all groups (r=0.114, p=0.025).

**(Table 1):**
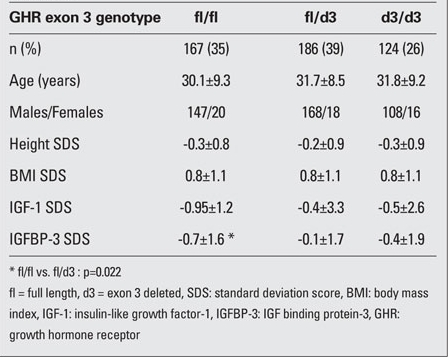
Table 1. Clinical and laboratory findings in different GHR exon 3 genotypes (mean±SD)

**(Table 2):**
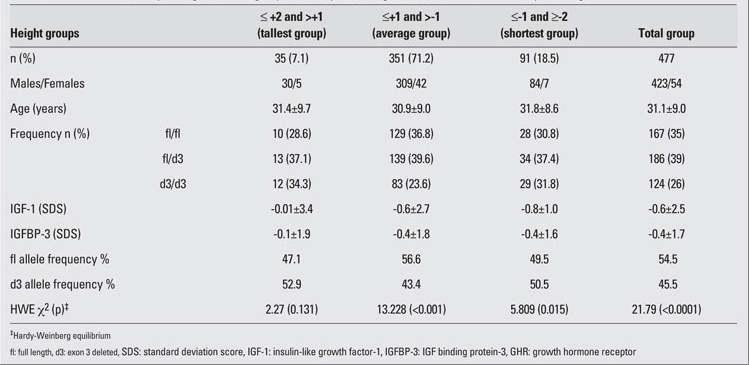
Table 2. Clinical and laboratory findings in the subgroups with respect to height SDS (mean±SD values and percentages)

## DISCUSSION

Adult height is not determined by a single gene but by complx polygenic inheritance. Studies in adult twins have shown that differences in height are related by 75-89% to genetic factors (18,19). Genetic influences on birth  length and postnatal growth are still being studied (20). Genome-wide analysis is expected to facilitate the  discovery of new genes related to somatic growth and to reveal the extent of impact of known genes. GHR exon 3 genotypes constitute the first genetic factor shown to affect the response to GH therapy (10). However,  subsequent studies about the extent of this effect have been inconclusive (3).  

Studies aiming to show the effects of GHR exon 3 genotypes on the height differences among populations revealed that the frequency of d3 allele was high among  the Caucasian population (8,11,21),  but low in Asian  populations (14,15,22).

 In our study, the frequencies of GHR exon 3 genotypes were 35% for fl/fl, %39 for fl/d3, 26% for d3/d3. The fl allele frequency was 54.5%, while the d3 allele frequency was 45.5%.  No difference was found in the distribution of GHR exon 3 genotypes among the shortest height group (height SDS between -1 and -2) and the other groups. Neither the total group nor any  height group, except for the tallest height group, reached HWE. GHR exon 3 genotype and allele frequencies were similar to those of European adults with normal stature and the homozygous d3 genotype was slightly more frequent (Table 3).

 Kenth et al (23) reported a GHR exon 3 genotype  distribution similar to our study, although they found a slightly higher (53.3%) fl/fl frequency and a slightly lower (11.1%) d3/d3 frequency. The authors reported an fl/d3  frequency of 35.9%. They failed to demonstrate an  association of adult height with BMI values and GHR  genotype. Raz et al (24) investigated GHR exon 3 genotype distribution in 211 Swedish adults with normal stature. The frequencies they estimated for the total group were 47.9% for fl/fl,  43.6% for fl/d3 and 8.5% for d3/d3. In addition, when the authors examined their sample by subgroups based on height, they found that d3/d3 frequency was  higher (13.1%) in the tallest subgroup (height SDS between +2 and +1) as compared to the total group and to the other subgroups. In comparison with the total group, d3 allele  frequency was slightly higher in the tallest height group (37% vs. 30%). In our study, d3/d3 genotype and d3 allele frequency were slightly higher in the tallest group, but this difference was not statistically significant.

 Audi et al (21) investigated GHR exon 3 genotype  distribution among 247 small for gestational age (SGA)  children and adolescents with short stature and among 289 adults with a height SDS between -2 and +2 SD. In this study, the frequency of genotypes in the adult group was reported as 27% for fl/fl, 57.8% for fl/d3 and 15.2% for d3/d3. The adults were divided into height subgroups, as tall (+2 and +1 SD), average height (+1 and -1 SD) and short (-1 and -2 SD). Among all adults, the estimated d3 and fl allele frequencies were 44% and 56%, respectively. Genotype distribution reached HWE in the total group, in the tall group and the short group.  In SGA children with short stature, the frequency of genotypes was fl/fl  44.2%, fl/d3 44.5%, d3/d3  11.3%; d3 allele  34%, and fl  allele 66%. The fl/d3 genotype was found to be more  common. In contrast, d3/d3 genotype frequency in shortest height group (fl/fl 25%, fl/d3 68.3%, d3/d3 6.7%, d3 allele frequency 41%, fl allele frequency 59%) was low. The  biologically less active fl/fl genotype was reported as twice more frequent in SGA patients with short stature. Compared to other studies conducted in Europe, the  reported frequency of fl/fl genotype in adults was slightly lower. Based on these findings, the authors concluded that d3/fl GHR exon 3 polymorphism may have an impact  on height.   

Millar et al (25) compared an African Beninese  population, which has a low mean height, and an English population in terms of GHR exon 3 genotype distribution. They observed that the Benineses had a significantly higher d3/d3 frequency (47% vs. 28%). Furthermore, d3/fl and fl/fl genotypes were also found to be higher among the Beninese than among the English population (70% vs. 47%). GHR exon 3 distribution did not reach HWE in either English or Beninese populations studied. The authors suggested that the higher incidence of GHR exon 3 deletion polymorphism among Benineses and the increase of GH1 expression and GHR-mediated growth response can be regarded as an adaptive response to famine and insufficient nutrition. 

In our study, although there was no difference among the genotype groups in terms of BMI SDS and IGF-1 SDS, the subjects with  heterozygous GHR exon 3 polimorphism had significantly higher IGFBP-3 SDS. There was a  significant positive correlation between BMI SDS and IGFBP-3 SDS. Recent studies have assessed the  interaction between efficacy of GH therapy and GHR exon 3 polymorphisms in various groups of GH deficient (GHD) patients (26,27,28). The results in GHD children are  conflicting. One study found a higher growth velocity in the group bearing at least one d3-GHR allele (26), whereas two other studies did not show this relationship (27,28). In the study of adult GHD patients, van der Klaauw et al (29) found a considerable increase in IGF-1 in patients bearing d3-GHR allele at short-term follow-up. In a study of 124 GHD adults, Barbosa et al (30) observed no influence of the d3-GHR allele on the response to GH therapy(31).

 The different results in the studies carried out in children and in adults highlight the risk of false negativity or positivity due to the interference of several factors on genotype-phenotype interactions. Only prospective and larger studies including a homogeneous group of GHD patients bearing all GHR  genotypes in a significant number, with similar inclusion and exclusion criteria as well as a similar therapeutic approach, would clarify the contribution of d3-GHR to the response to GH therapy. 

In conclusion, this study presents the results of GHR  polymorphism in a Turkish population as a reference for  further studies. The distribution was similar to that in European populations. There were no correlations between heights of the individuals and GHR isoforms except for a  higher IGFBP-3 in the fl/d3 group as compared to the fl/fl group. 

**Table 3:**
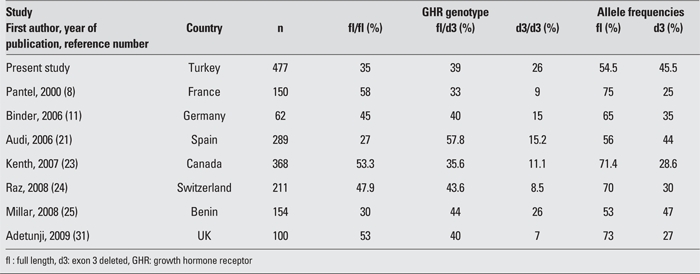
Table 3. The distribution of GHR exon 3 genotypes in different populations
